# Preliminary Study on Biosynthesis of Bacterial Nanocellulose Tubes in a Novel Double-Silicone-Tube Bioreactor for Potential Vascular Prosthesis

**DOI:** 10.1155/2015/560365

**Published:** 2015-05-19

**Authors:** Feng Hong, Bin Wei, Lin Chen

**Affiliations:** ^1^Group of Microbiological Engineering and Industrial Biotechnology, College of Chemistry, Chemical Engineering and Biotechnology, Donghua University, North Ren Min Road, No. 2999, Songjiang, Shanghai 201620, China; ^2^State Key Laboratory for Modification of Chemical Fibers and Polymer Materials, Donghua University, Shanghai 201620, China

## Abstract

Bacterial nanocellulose (BNC) has demonstrated a tempting prospect for applications in substitute of small blood vessels. However, present technology is inefficient in production and BNC tubes have a layered structure that may bring danger after implanting. Double oxygen-permeable silicone tubes in different diameters were therefore used as a tube-shape mold and also as oxygenated supports to construct a novel bioreactor for production of the tubular BNC materials. Double cannula technology was used to produce tubular BNC via cultivations with *Acetobacter xylinum*, and Kombucha, a symbiosis of acetic acid bacteria and yeasts. The results indicated that Kombucha gave higher yield and productivity of BNC than *A. xylinum*. Bacterial nanocellulose was simultaneously synthesized both on the inner surface of the outer silicone tube and on the outer surface of the inner silicone tube. Finally, the nano BNC fibrils from two directions formed a BNC tube with good structural integrity. Scanning electron microscopy inspection showed that the tubular BNC had a multilayer structure in the beginning but finally it disappeared and an intact BNC tube formed. The mechanical properties of BNC tubes were comparable with the reported value in literatures, demonstrating a great potential in vascular implants or in functional substitutes in biomedicine.

## 1. Introduction

Large vascular applications of vascular prosthesis (>6 mm in diameter) as a substitute have been clinically realized. Synthetic materials have been used to make artificial blood vessels for replacement, among which expanded polytetrafluoroethylene (ePTFE) and polyethylene terephthalate (PET) are the most acceptable. However, the problem with the reactivity between the components in the blood and the internal surface of the materials is still stubborn. For small vessel (<6 mm in diameter), which occupies the majority of blood vessels in the body, a substitute has not been well developed, especially in bypass surgery. It is known that the small vascular grafts made of ePTFE and PET material would often result in heavy problems such as the formation of a thrombus before the formation of the pseudo living body structure when implanted for period of time. The thrombus blocks the flow of blood stream or causes cerebral thrombosis, myocardial infraction, and pulmonary infraction, which leads to a fatal crisis occurring in the human body [1]. The current standard is to use autologous minute vessels such as saphenous veins or internal mammary arteries. Therefore, there is a growing demand to find and develop succedaneous materials for small-diameter blood vessels.

Bacterial nanocellulose (BNC, also called bacterial cellulose, BC) is a kind of natural cellulose polymer synthesized by some microorganisms, for example,* Acetobacter xylinum* (currently* Gluconacetobacter xylinus*, belonging to Gram-negative bacteria) [2] and Kombucha (also called “black tea fungus,” which is composed of a kind of symbiotic microbial communities mainly containing acetic acid bacteria and yeasts) [3–7]. Although chemically identical to plant cellulose, BNC is characterized by a unique fibrillar nanostructure which determines its distinguished physical and mechanical properties, including high porosity, high wet tensile strength, high water-holding capacity, and good biocompatibility [2, 8]. Therefore, BNC has been recognized as a promising natural biomedical material with high potential for artificial blood vessels [9–20], wound dressing [21–27], and tissue engineering materials [28–30].

**Figure 1:**
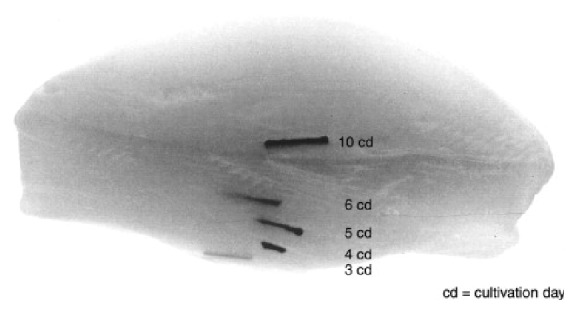
Formation process of BNC layers during 10 cultivation days displayed by marking experiments [11].

**Figure 2:**
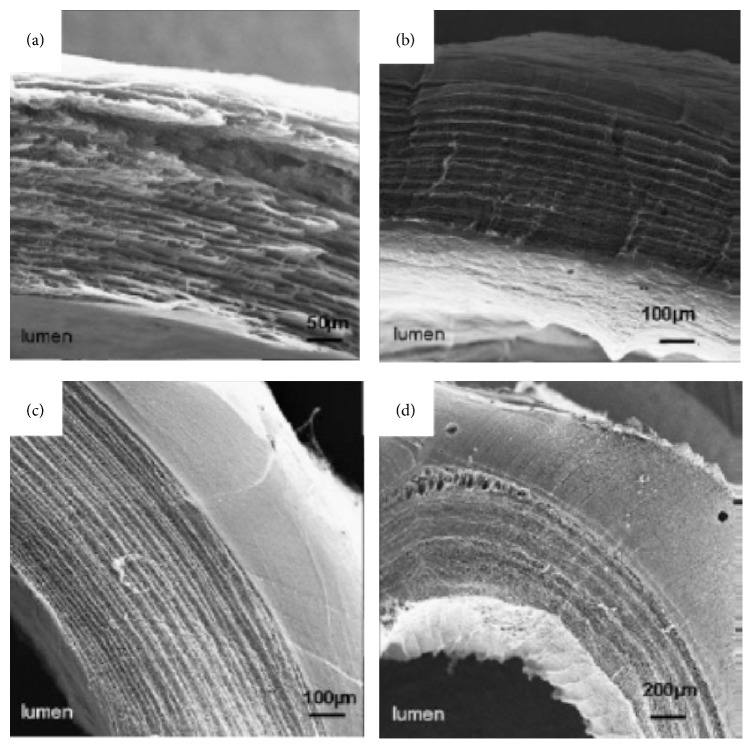
Scanning electron microscope images of BNC tubes obtained with the second device [14].

More recently, tubular BNC material has attracted increasing attentions since it has a great potential as artificial blood vessels, especially small vascular grafts. Despite being produced by the Gram-negative bacteria, the material is purified successfully with sodium hydroxide with endotoxin values in respect to American FDA for implants in contact with blood, that is, <20 EU per device. Several researches have showed that BNC is biocompatible, probably because of the similarity between the porous and flexible network of cellulose microfibrils to collagen. It has high mechanical strength due to fibril entanglements and a high water content that prevents proteins to adsorb [11, 13, 14, 28]. The BNC can be manufactured* in situ* in various sizes and shapes via fermentation depending on the product requirements, and some efforts have been made to prepare tubular BNC materials by designing various cultivation devices. There are basically three kinds of cultivation devices to prepare BNC tubes. The first kind of device consists of a vertically placed tubular glass vessel and a glass stick or tube fixed in the center. A short BNC tube can be formed between the gaps in the device when inoculated culture media are filled in and statically incubated for 7–14 days. Yamanaka et al. [10] and Klemm et al. [11, 12] successfully prepared tubular BNC materials in this device. But this method spends relative long cultivation time and it is very difficult to produce a BNC tube longer than 20 mm (normally 15–20 mm) since the length is restricted by the thickness of BNC pellicle. The second one contains an oxygen-permeable silicone tube as a support together with a gas inlet through the tube, which is placed in a cultivation container that is filled with inoculated culture media. A BNC tube forms on the surface of the silicone tube after cultivation for several days. Yamanaka et al. [10], Bodin et al. [14], Putra et al. [16], Jia et al. [18], and Bäckdahl et al. [19] used this kind of device to produce BNC tubes with desired length. However, the obtained BNC tubes were very thin and their mechanical performance was not satisfactory. In the third device, a glass rod is fixed horizontally with rubber rings in the core of a tubular cultivation vessel consisting of two half-pipes. The device is placed on the surface of a precultured bacterial cellulose pellicle. During the continued-cultivation, the newly generated nanocellulose will continue to grow into the cultivation vessel through the gap between the two half-pipes and finally fills the whole space of the vessel. The formed BNC material in the vessel is a tubular material. Bertholdt [15] obtained a long BNC tube successfully by using this device. This method is able to provide a BNC tube in different size, especially for the long tube within a short incubation time as compared to the first kind of device. A main common disadvantage of the BNC tubes produced by using the three kinds of devices described above is that the BNC tubes show a multilayer structure because of an inherent feature (accumulation layer by layer during cultivation) in the BNC material obtained in static cultures (Figure 1 [11] and Figure 2 [14]).

In this study, an integral tubular BNC material without the layered structure was prepared by using a patented novel bioreactor composed of double silicon tubes [31]. The synthesized tubular BNC materials had desirable properties such as uniform thickness, controllable length, and small inner diameter of less than 5 mm. The obtained tubular BNC material was characterized by field emission scanning electron microscopy and attenuated total reflection Fourier transform infrared spectroscopy, and its mechanical properties were determined by measurements of tensile strength, burst pressure, and suture retention in the preliminary study.

Although the appearance of the proposed double-silicone-tube bioreactor in the study looks similar to that of the first kind of device as mentioned above, actually they are completely different. The first kind of bioreactor comprises double glass tubes just serving as a mold to shape the cellulose pellicle that is formed in conventional static cultures, and it is difficult to get a BNC tube longer than 20 mm. Our device can produce a much longer BNC tube as long as the length of silicon tubes provided in the reactor is long enough. The second kind of reactor consists of single silicone tube but not double silicone tubes applied in the study. The formation mechanism of BNC in our bioreactor is the same as that in the second kind of bioreactor because BNC forms on the surface of silicone tube. However the production efficiency in our bioreactor should be faster. This is because BNC only forms on the single silicone tube in the second kind of bioreactor but BNC shapes from two directions in our bioreactor, namely, at the double-silicone-tube-mode.

## 2. Materials and Methods

### 2.1. Microorganisms and Chemicals

Kombucha was purchased from Heilongjiang province and maintained at 4°C.* Acetobacter xylinum* (currently* Gluconacetobacter xylinus* ATCC 23770) was obtained from American Type Culture Collection (Manassas, VA) and maintained on agar plates containing a seed culture medium, which consisted of (w/v) 2.5% D-mannitol, 0.5% yeast extract (Oxoid, UK), 0.3% peptone, and 2.5% agar at initial pH 5.0.

The fermentation medium used for BNC production contained (w/v) is as follows: 0.5% green tea, 10% glucose, 0.3% tryptone, and 0.5% yeast extract. Prior to pasteurization the pH value of the medium was not adjusted.

### 2.2. Preparation of Preculture

For Kombucha, a piece of BNC pellicle from Kombucha culture was transferred into a 250 mL Erlenmeyer flask containing 50 mL fermentation medium mentioned above and then incubated statically at 30°C to activate the culture for 7 days. Two pieces of the harvested Kombucha BNC pellicle in 5 mm diameter were transferred into the fermentation medium of 50 mL. For* Acetobacter xylinum*, two loops of the strain were picked up from an agar plate and were inoculated into a conical flask containing 50 mL fermentation medium mentioned above. After cultivation at 30°C and 160 rpm on a rotary shaker for 12–24 h, a fermentation broth was ready for production of BNC tubes.

### 2.3. Production of BNC Tubes

**Figure 3:**
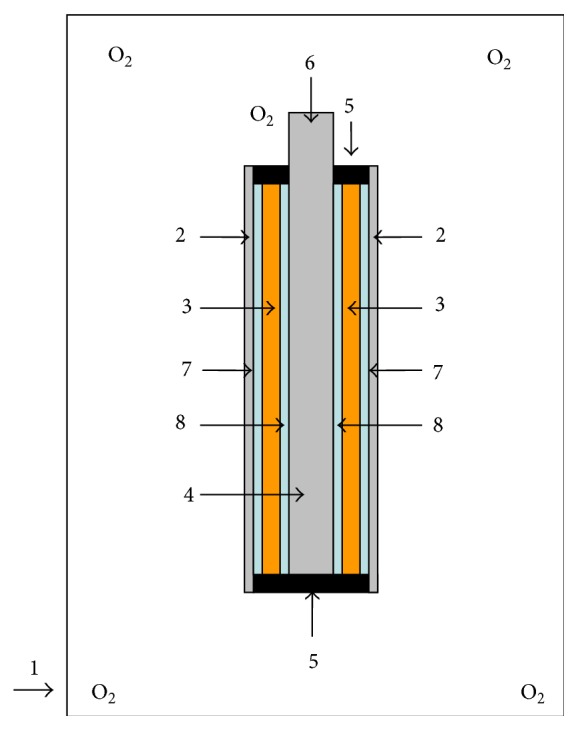
Schematic diagram of the bioreactor for production of tubular BNC. (1) Airtight container full of oxygen, (2) outer silicone tube, (3) fermentation broth, (4) inner silicone tube, (5) hollow cylindrical silicone plug, (6) inlet of oxygen, (7) BNC layer synthesized on the inner surface of the outer silicone tube, and (8) BNC layer synthesized on the outer surface of the inner silicone tube.

Two oxygen-permeable silicone tubes in different diameters and lengths were used as a mold and then a novel bioreactor (see Figure 3 [31]) was constructed for production of BNC tubes as potential artificial blood vessels. In the bioreactor, the outer silicone tube possessed an external diameter of 9 mm and a length of 60 mm, while the inner silicone tube was 3 mm and 80 mm, respectively. Both silicone tubes had the same wall thickness of 0.5 mm.

After being washed and dried, the silicone tubes were sterilized in an autoclave at 121°C for 20 min and fixed axisymmetrically together with two hollow cylindrical silicone plugs in the same size (component 5) to construct the bioreactor (Figure 3). After that, double cannula technology was used to produce BNC tubes via bacterial fermentation. The precultures of* Acetobacter xylinum* and Kombucha were transferred into the interspace between the two fixed tubes of the bioreactor, respectively. The bioreactor was then incubated statically at 30°C for 4–7 days in an airtight container full of oxygen. During the incubation, bacterial cellulose was synthesized and formed both on the inner surface of the outer silicone tube and on the outer surface of the inner silicone tube simultaneously. Finally, the nano BC fibrils from the two directions were interweaved and an integrative bacterial nanocellulose tubular material formed.

### 2.4. Purification of BNC Tubes

The harvested BNC tubes were washed with distilled water to remove medium components and then incubated in 1.0% NaOH aqueous solution at 80–90°C for 2 h to eliminate attached cells. After that, the BNC tubes were further purified to remove other impurities with distilled water until the pH of the washing liquid was neutral.

### 2.5. Characterization of BNC Tubes

#### 2.5.1. Morphology

The morphology and microstructure of BNC tubes were examined by using field emission scanning electron microscopy (FESEM, HITACHI-S4800). Prior to observation, small segments of the BNC tubes were freeze-dried and sputter-coated with carbon. The images were obtained at an acceleration voltage of 5.0 kV.

#### 2.5.2. ATR-FTIR Analysis

The BNC tubes produced by Kombucha and* Acetobacter xylinum* were analyzed by attenuated total reflectance Fourier transform infrared (ATR-FTIR) spectroscopy and the spectra were recorded on a Fourier transform spectrophotometer (NICOLET NEXUS 670) in the range from 4000 to 500 cm^−1^. All the samples were freeze-dried for 1 day before analysis.

#### 2.5.3. Tensile Strength Measurement

A universal material testing machine (H5KS, Hounsfield Co. Ltd.) was used to measure the mechanical properties of the tubular BNC at room temperature according to [16, 28]. The BNC tubes of 40 mm in length were stretched in two directions, that is, lengthwise (axial direction) and breadthwise (radial direction). For lengthwise stretching, the sample was fastened in a clip, leaving the length of 10 mm from both of the edges. For breadthwise stretching, two steel wires of U-shape were inserted through the BNC tube and fastened in the clips, respectively. All the samples were stretched at a velocity of 100 mm/min. In all the tests, stretching measurements were performed in triplicate.

#### 2.5.4. Burst Pressure Measurement

Burst pressure was measured by home-made equipment which was equipped with a pressure gage and a syringe. BNC tube sample with a size of 40 mm in length was well connected to the equipment. PBS buffer was injected into the tube at a speed of approximately 0.01 MPa/s (75 mm Hg/s) until failure. The pressure in the tube was kept for 10 s at every 0.02 MPa. The pressure was recorded at the point of sudden burst of the tube. Each test was performed three times.

#### 2.5.5. Suture Retention Measurement

Suture retention was measured by a single throw of 6–0 prolene suture through the BNC tube fixed to the stage clamp of the universal material testing machine. The suture was placed in the four corners 2 mm from the edge of the tube. The tube was pulled at a constant rate of 100 mm/min until the suture pulled through the BNC tube. The strength at failure was recorded. Each test was performed three times.

## 3. Results and Discussion

### 3.1. Morphology of BNC Tubes

**Figure 4:**
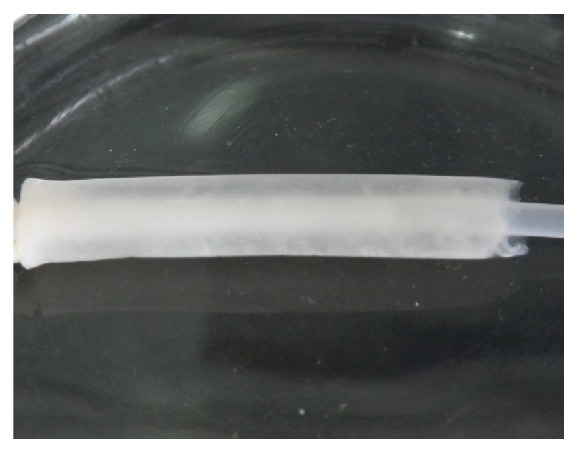
Photo of tubular BNC material produced in the double-silicone-tube bioreactor inoculated with Kombucha.

It was reported that the oxygen permeability of the tubular mold was crucial for aerobic bacteria to proliferate and synthesize BNC material. Silicone tubes have been proved to be a suitable oxygenated support for formation of BNC [14–19]. In our study, in order to improve the oxygen supply to enhance bacterial growth and BNC formation, two oxygen-permeable silicone tubes in different diameters were combined as a tubular mold to provide a space between the external big tube and the interior small tube. One of the sides of each of the tubes can touch oxygen as double oxygenated supports. The bacterial cultivation was performed in the interspace of the two fixed silicone tubes. During the cultivation, BNC was found to be synthesized and formed simultaneously on both the inner surface of the external big silicone tube and the outer surface of the interior small silicone tube in the bioreactor, especially in the first 3 days of cultivation. Subsequently, the two parts of cellulose fibrils interwove with each other and became an intact tubular material after 7-d incubation. The internal diameter, wall thickness, and length of the obtained BNC tubes were 3 mm, 2.5 mm, and 45 mm, respectively, which was dependent on the shape of the interspace between the silicone tube molds (Figure 3). The BNC tubes produced by Kombucha were shown in Figure 4. Both the inner and outer surfaces of the tubular BNC material were smooth and uniform, which would benefit its application as vascular graft since it was found that uniform surface was very important for avoiding the formation of thrombus [10–12, 17].

This study is a preliminary investigation with the aim to show that it is feasible to use the designed double-tube reactor to make the BNC vessel more efficiently. Therefore only one size of the silicone tube was used as a mode to prove the proposed technology and bioreactor as well as to demonstrate the successful preparation of the BNC tube. The reason why only 45 mm was obtained is because that the two hollow cylindrical silicone plugs (component 5 in Figure 3) occupied some spaces of the bioreactor of 60 mm in length. It is no problem to apply the promising technology with the new designing concept to construct other bioreactors with double tubes in different sizes. More bioreactors constructed with silicone tubes in various sizes will be investigated to make the BNC tubes with other dimensions in further studies.

Additionally, in our experiment, the strain of* A. xylinum*, which is often used in the BNC study, was also utilized to synthesize tubular BNC materials under the same conditions. However, it spent 25 days to achieve the same wall thickness and length of the BNC tube as those from Kombucha. Therefore, application of Kombucha in the biosynthesis of BNC tubes could efficiently shorten the incubation time because of the synergy of symbiotic microbes as compared to the* A. xylinum*. As far as we know, our study was the first time to utilize Kombucha to produce BNC tubes. Many factors including culture media and cultivation conditions would affect the growth of the strain of ATCC 23770 and the formation of cellulose. For instance, concentration of sugar and oxygen would remarkably influence the formation of BNC with the ATCC 23770. Optimization of the cultivation has not been performed so far but will be done in further studies. It is a fact that the clear definition of the microbial strain is the ultimate prerequisite for the essential reproducibility of medical devices. Some works on isolation of potent bacterial strains from the Kombucha are being performed and several strains were found to be capable of making BNC tubes in the double-tube reactor efficiently. These results will be introduced in other manuscripts in future.

**Figure 5:**
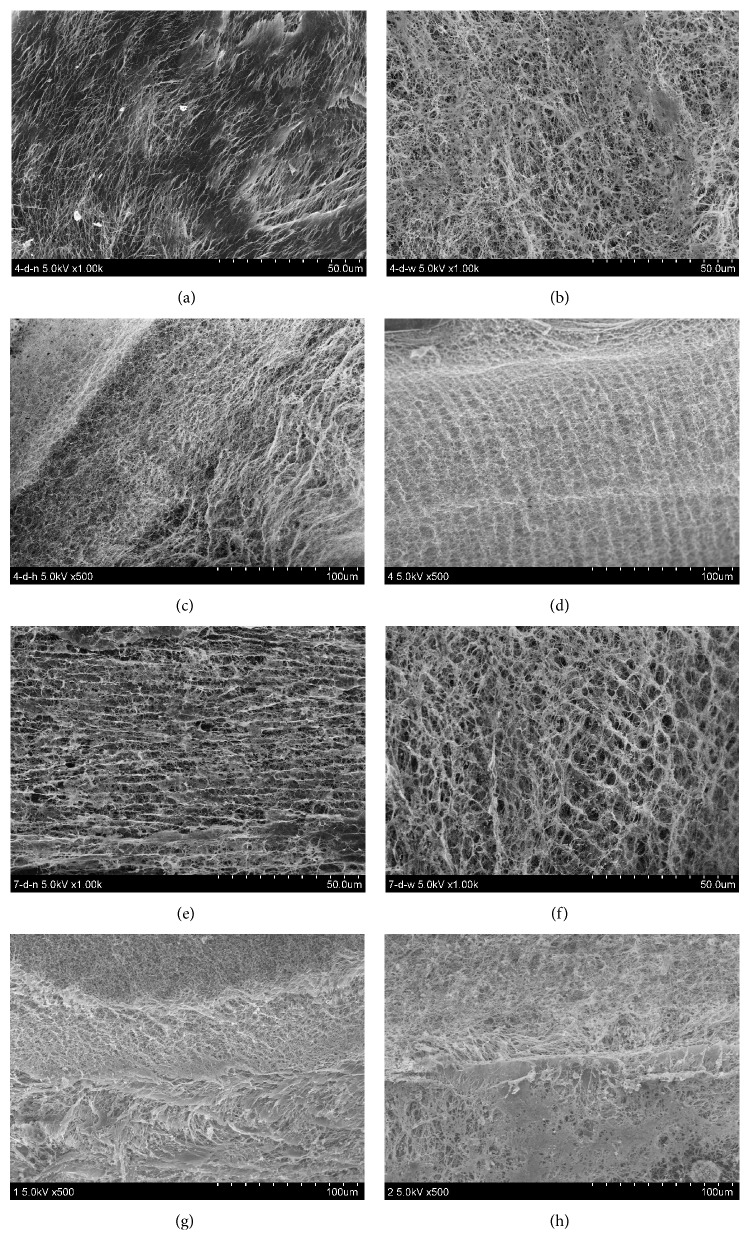
FESEM images of tubular BNC materials. (a)–(d) The images of BNC tube obtained after 4-d cultivation. (a) The inner surface of the BNC tube with magnification at 1000x; (b) the outer surface of the BNC tube with magnification at 1000x; (c) the cross section of the BNC tube with magnification at 500x; (d) longitudinal section of the BNC tube with magnification at 500x. (e)–(h) The images of BNC tube obtained after 7-d cultivation. (e) The inner surface of the BNC tube with magnification at 1000x; (f) the outer surface of the BNC tube with magnification at 1000x; (g) cross section of the BNC tube with magnification at 500x; (h) longitudinal section of the BNC tube with magnification at 500x.

**Figure 6:**
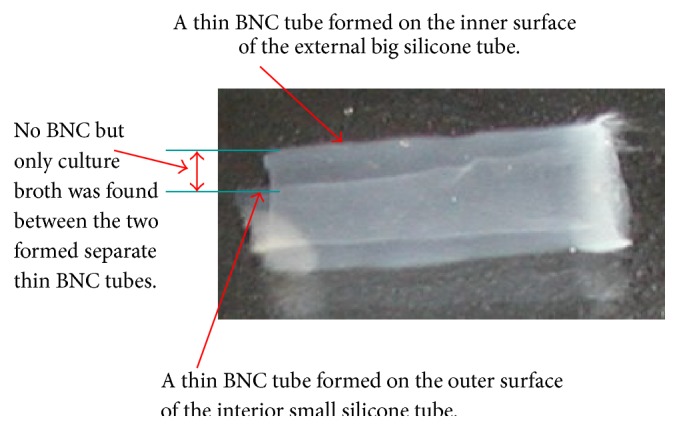
Image of a BNC sample obtained on the third day.

The FESEM images display the microstructure of BNC tubes (as shown in Figure 5). Figure 5 shows that all the BNC tubes have denser inner and outer surface (Figures 5(a)-5(b) and 5(e)-5(f)), and the BNC network structure shows proper fibril orientation. It is noteworthy that the structures of the inner and outer surface of the BNC tube are different (Figures 5(a) to 5(b) and 5(e) to 5(f)). The reason behind the finding is not clear. Although both BNC formed on silicone tubes, probably the fine structure of the silicone surface and the different oxygen-permeable area of the silicone tubes would affect the structure of the BNC formed on the silicone touching surface. It is well-known that oxygen supply is able to influence the generation and structure of BNC. This needs further study. During the initial cultivation, such as 4-d cultivation, all the tubes comprise a layered structure, which can be observed on the cross and longitudinal sections of the tubes (Figures 5(c) and 5(d)). Significant difference in density was observed in the 4-d incubation (Figure 5(c)); however interestingly, the density of BNC tubes increased with an increase of incubation time, and finally no significant layered structure could be found (Figure 5(g)). An image shows that two split thin BNC tubes were found and no integral vessel formed before 4 days of cultivation (Figure 6). This observation validated the assumption that two parts of BNC fibrils formed simultaneously on both the inner surface of the external big silicone tube and the outer surface of the interior small silicone tube, subsequently interwove slowly with each other, and then finally became one intact tubular material.

The initial layered structure also can be found when BNC is formed in the conventional static culture with the single silicon tube mold, as described in previous studies [10–12, 14–19]. However, our technology obtained a more favorable intact BNC tube without significant layered structure after 7-d cultivation. That BNC layers interwove with each other to form an intact material might benefit the mechanical property of the BNC tubes as compared to those BNC tubes formed on the single silicone tube mold.

### 3.2. Analysis of BNC Tubes by ATR-FTIR

**Figure 7:**
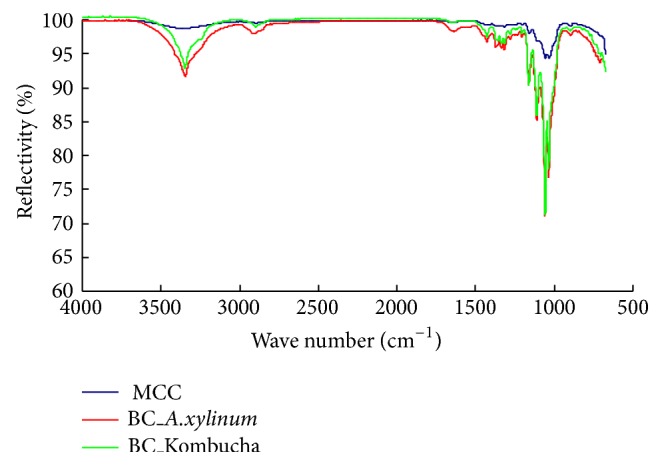
ATR-FTIR analyses of various cellulose materials. Legends of MCC, BC_A.xylinum, and BC_Kombucha are microcrystalline cellulose, the BNC tube produced by* A. xylinum* and the BNC tube produced by Kombucha, respectively.

ATR-FTIR analysis of BNC samples that were synthesized with Kombucha and* A. xylinum* was compared and the spectra are illustrated in Figure 7. Microcrystalline cellulose (MCC) was used as a control in the analysis. The results showed that all the ATR-FTIR spectra of BNC_A.xylinum, BNC_Kombucha, and MCC had four characteristic peaks of cellulose from 900 to 1300 cm^−1^. The band at 1160 cm^−1^ was assigned to the C–O–C asymmetric stretching and the peaks at 1313 and 1426 cm^−1^ were attributed to CH_2_ wagging symmetric bending and CH_2_ symmetric bending, respectively. The bands present between 3200 and 3400 cm^−1^ corresponded to O–H stretching modes in alcoholic groups. The appearance of the narrow peaks from 1300 to 1800 cm^−1^ might attribute to the residues from culture medium because of incomplete washing for thick BNC pellicles. From the spectra, it could be concluded that the BNC materials synthesized by Kombucha and* A. xylinum* showed no significant difference in chemical composition.

### 3.3. Tensile Strength Measurement of BNC Tubes

**Figure 8:**
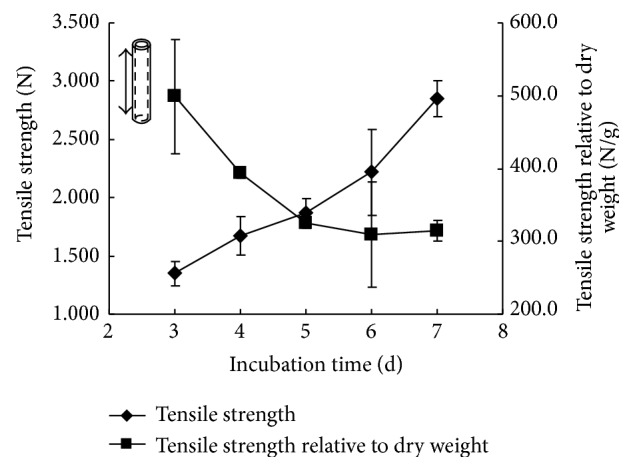
Effect of incubation time on tensile strength of BNC tubes in lengthwise stretching.

**Figure 9:**
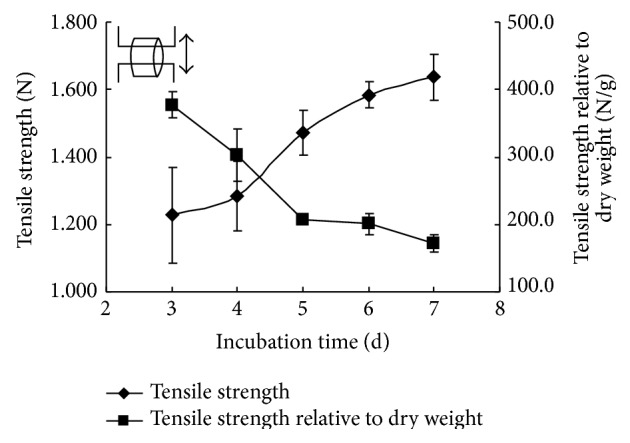
Effect of incubation time on tensile strength of BNC tubes in breadthwise stretching.

Incubation time of BNC tubes had a remarkable effect on its mechanical properties. BNC tubes were stretched in two directions, lengthwise and breadthwise. The curves of tensile strength and tensile strength relative to dry weight are shown in Figures 8 and 9. BNC looks like a hydrogel, and it is soft and easily deformed, which means that it is not easy to measure the accurate size of BNC tube when it is in a highly swollen wet state. Since BNC usually contains 99% water and will give significant change in wall size due to much loss of water during stretching, the conversional unit of Pascal for characterization of tensile strength was therefore not adopted here and a unit of Newton for absolute force was applied instead.

The results showed that the tensile strength of tubular BNC in both lengthwise and breadthwise directions increased with the prolonged incubation time. The main reason might be ascribed to the higher density in BNC tube wall achieved in longer cultivation. It was in agreement with the structural observation by FESEM that the density of BNC tubes increased with the extended incubation time because network structures became dense or compact. Furthermore, the tensile strength relative to dry weight showed a similar decline tendency in both lengthwise and breadthwise stretching. This should be attributed to that the increase rate of tensile strength was lower than that of dry mass of BNC formed in both lengthwise and breadthwise directions. The maximum tensile forces in lengthwise and breadthwise stretching reached up to approximately 2.8 N (Figure 8) and 1.6 N (Figure 9), respectively. These values reflect that the load capacity of obtained BNC tubes in our study is comparable to the reported value of 0.8 N in the BNC tube obtained in the first kind of incubation device and also comparable to those in native tissue blood vessels (0.356–2.21 N) [11].

### 3.4. Measurement of Burst Pressure and Suture Retention of BNC Tubes

Burst pressure strength is one of the most important indices for examining the mechanical properties of vascular implants. To determine whether the BNC vessels possessed adequate strength to endure physiological forces, burst pressure testing was performed to identify the maximum pressure that the vessels could endure before failure. The burst pressure strength of the BNC vessels was 29.2 ± 3.4 kPa (*n* = 3, *P* > 0.05). Because a normal blood pressure in human body is in the range of 12.0–18.7 kPa (90–140 mm Hg) [32], the results indicate that the BNC tubes possessed adequate physical strength and could be developed as substitutes for native blood vessels.

Suture retention strength is a crucial factor in the handling characteristics of vascular prosthesis, as it directly determines the success of the vascular graft implantation procedure. The suture retention strength of 10 pieces of wet BNC tubes in highly swollen state was 0.49 ± 0.02 N (*P* = 0.34). The *P* value shows no significant difference among the 10 samples (*P* > 0.05). Although the suture retention strength was somewhat low since the tubes contained too much water (>98%), the suture strength of the BNC tubes is comparable to the reported values (47 g, namely, 0.46 N) of fresh human tissue-engineered vessels [33]. The vessels with low strength were suggested to be anastomosed to the native aorta without anastomotic bleeding or tearing at the suture line [33]. After dehydration with absorption of tissue paper to reach a wall thickness of 1.3 mm, the suture strength of BNC vessels was improved significantly to 1.62 ± 0.03 N. This means that the dehydrated BNC tubes had adequate suture retention strength for suturing during implantation, which is generally accepted to be 1.0 N [34]. As suggested by Dr. Klemm, this kind of BNC tubes can be used as substitutes of tubular organ for surgery training in medical colleges [11].

## 4. Conclusions 

A novel bioreactor equipped with two oxygen-permeable silicone tubes as mold supports in different diameters has been successfully constructed. It was the first time to utilize Kombucha as a production culture to biosynthesize tubular BNC materials and subsequently shorten the incubation time efficiently. The preliminary results indicated that the nano BC fibrils from two directions should be interwoven together, and finally an intact BNC tubular material was obtained with desirable mechanical properties but without the layered structure observed in previous studies. Both the inner and outer sides of the tubular BNC material were smooth and uniform. Its mechanical properties were comparable with the reported values in literatures, and the BNC tubes own a great potential in vascular implants or in other functional substitutes in biomedicine, for example, artificial hollow organ including ureter and esophagus. The proposed production technology of the tubular BNC material is quite simple, and accordingly other hollow BNC materials in different shapes could also be produced on the basis of the introduced concept. More further investigations on biocompatibility, hemocompatibility, compliance, and biosafety as implants and how to regulate the structure and mechanic properties of BNC tubes are underway.
